# Modulation of the Biocatalytic Properties of a Novel Lipase from Psychrophilic *Serratia* sp. (USBA-GBX-513) by Different Immobilization Strategies

**DOI:** 10.3390/molecules26061574

**Published:** 2021-03-12

**Authors:** Mónica Ruiz, Esteban Plata, John J. Castillo, Claudia C. Ortiz, Gina López, Sandra Baena, Rodrigo Torres, Roberto Fernandez-Lafuente

**Affiliations:** 1 Escuela de Química, Grupo de investigación en Bioquímica y Microbiología (GIBIM), Edificio Camilo Torres 210, Universidad Industrial de Santander, Bucaramanga 680001, Colombia; icmonicaruiz@gmail.com (M.R.); stbnplata29@gmail.com (E.P.); jcasleon@uis.edu.co (J.J.C.); 2 Escuela de Microbiología, Universidad Industrial de Santander, Bucaramanga 680001, Colombia; ortizc@uis.edu.co; 3 Unidad de Saneamiento y Biotecnología Ambiental (USBA), Departamento de Biología, Pontificia Universidad Javeriana, Bogotá 11001000, Colombia; ginalopezr@gmail.com (G.L.); baena@javeriana.edu.co (S.B.); 4 Laboratorio de Biotecnología, Instituto Colombiano del Petróleo, ECOPETROL, Piedecuesta 681012, Colombia; rodrigo.torres@ecopetrol.com.co; 5 Departamento de Biocatálisis—ICP-CSIC, Campus UAM-CSIC Cantoblanco, 28049 Madrid, Spain; 6 Center of Excellence in Bionanoscience Research, External Scientific Advisory Academic Board, King Abdulaziz University, Jeddah 21589, Saudi Arabia

**Keywords:** lipase from psychrophilic microorganism, immobilization, interfacial activation, hyperactivation, USBA-GBX-513

## Abstract

In this work, the effect of different immobilization procedures on the properties of a lipase obtained from the extremophilic microorganism *Serratia sp*. USBA-GBX-513, which was isolated from Paramo soils of Los Nevados National Natural Park (Colombia), is reported. Different Shepharose beads were used: octyl-(OC), octyl-glyoxyl-(OC-GLX), cyanogen bromide (BrCN)-, and Q-Sepharose. The performance of the different immobilized extremophile lipase from *Serratia* (ESL) was compared with that of the lipase B from *Candida antarctica* (CALB). In all immobilization tests, hyperactivation of ESL was observed. The highest hyperactivation (10.3) was obtained by immobilization on the OC support. Subsequently, the thermal stability at pH 5, 7, and 9 and the stability in the presence of 50% (*v*/*v*) acetonitrile, 50% dioxane, and 50% tetrahydrofuran solvents at pH 7 and 40 °C were evaluated. ESL immobilized on octyl-Sepharose was the most stable biocatalyst at 90 °C and pH 9, while the most stable preparation at pH 5 was ESL immobilized on OC-GLX-Sepharose supports. Finally, in the presence of 50% (*v*/*v*) tetrahydrofuran (THF) or dioxane at 40 °C, ESL immobilized on OC-Sepharose was the most stable biocatalyst, while the immobilized preparation of ESL on Q-Sepharose was the most stable one in 40% (*v*/*v*) acetonitrile.

## 1. Introduction

Most of the soluble enzymes are not suitable for industrial applications where harsh conditions, such as high temperatures or the presence of organic solvents, are generally required [1,2]. For this reason, immobilization techniques have emerged as an alternative to improve the catalytic properties and the operational stability of the enzymes, allowing enzymatic processes to be cost-effective compared to chemical ones. A proper immobilization protocol may enhance enzyme stability, activity, selectivity, or specificity, and even enzyme purity [3,4,5,6,7,8,9,10,11,12,13].

Lipases are the most used enzymes in biocatalysis due to their high activity, specificity, selectivity, and robustness in a variety of reaction media [14,15,16,17,18,19,20,21,22,23,24]. Lipases have a peculiar catalytic mechanism, called interfacial activation, which is based on the interaction of the lipase with hydrophobic structures, which causes deep conformational changes on the structure of the active site of the lipase and its consequent enzyme activation [25,26,27,28,29], permitting the enzyme to act in the hydrolysis of insoluble drops of oils and be adsorbed on any hydrophobic surface [30,31,32,33,34].

A significant number of reversible and irreversible immobilization protocols have been developed to immobilize lipases [12,31,34,35,36,37,38,39]. Such immobilization strategies can improve enzyme stability and even the enantioselectivity or enantiospecificity of the biocatalyst if properly performed [16]. Additionally, proper selection of the type of support and immobilization protocol is important to achieve optimal results [40]. One of the most used strategies in the immobilization of lipases is their immobilization on hydrophobic supports via lipase interfacial activation. This immobilization protocol has the following advantages [34]: specific lipase immobilization (which facilitates its purification in most cases) [6,34]; fixation of the open form configuration of the lipase, causing hyperactivation and stabilization of the enzyme and modulation of their enzyme properties; and easy to perform and reversible, which allows the immobilization support to be reused [41]. For instance, Urrutia et al. immobilized lipase B from *Candida antarctica* (CALB) and lipase from *Rhizomucor miehei* (RML) on a chitosan support functionalized with different alkyl chains (C4, C8, C12) and used the biocatalysts for the selective hydrolysis of fish oil, obtaining a higher expressed activity at a longer alkyl chain length modification of the support [42]. The main problem of this lipase immobilization strategy is the risk of releasing enzyme molecules from the support under certain conditions [43]. This may be solved using acyl-heterofunctional supports [44,45,46] or crosslinking the enzymes (physically or covalently) [47].

On the other hand, the demand for enzymes that can resist harsh conditions for industrial processes has encouraged the search for enzymes obtained from microorganisms that live in extreme conditions and non-conventional environments (e.g., pH, salinity, temperature, pressure, or radiation) [48,49,50,51,52]. These microorganisms, called extremophiles, produce enzymes capable of catalyzing reactions under non-conventional conditions [53,54,55]. Thermophilic enzymes like extremophile proteases, lipases, and polymer-degrading enzymes have found applications in the food and cosmetic industries [56,57,58]. Very interestingly, the advantages of using thermophilic enzymes may be coupled with the use of immobilization technologies to further improve enzyme features [59].

**Figure 1:**
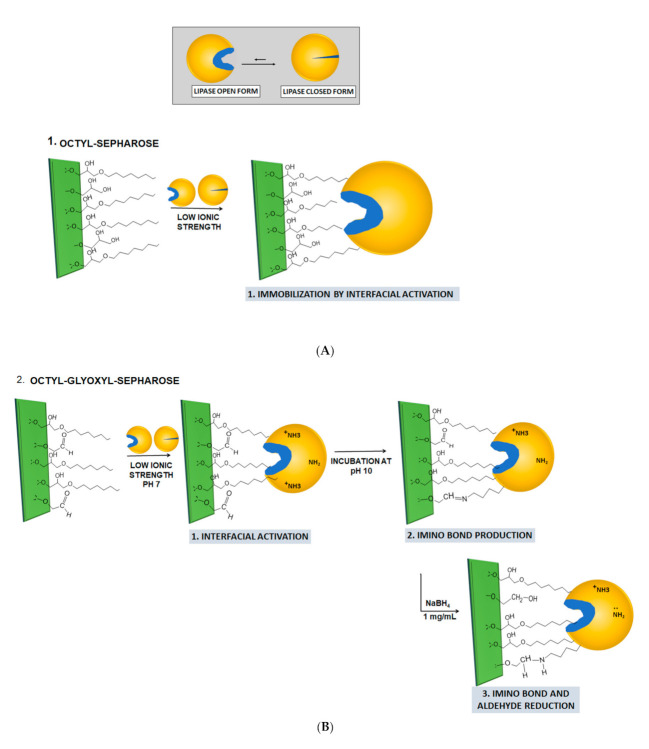

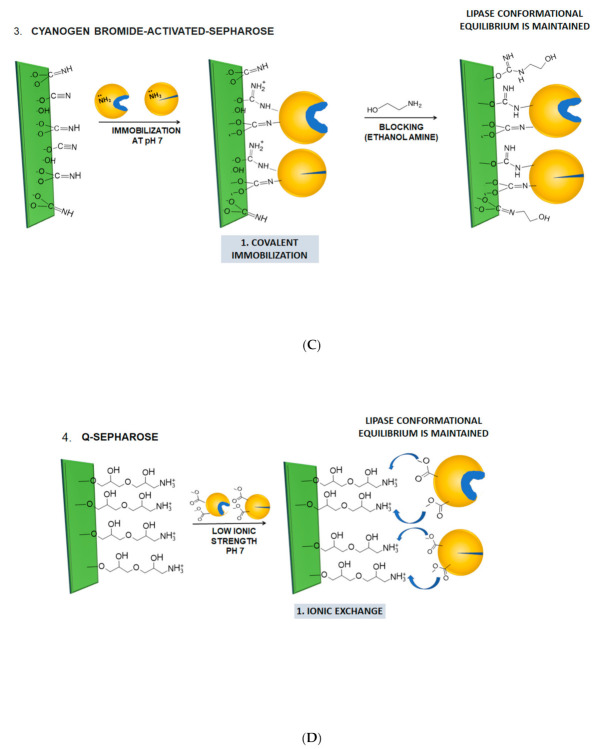
Different immobilization protocols used in this research. (**A**): Immobilization on octil-Sepharose; (**B**): Immobilization on octyl-glyoxyl Sepharose; (**C**): Immobilization in BrCN-Sepharose; (**D**): Immobilization in Q-Sepharose.

Recently, the lipase from *Serratia* sp. (USBA-GBX-513), a tolerant psychrophilic bacterium, was isolated from Paramo soils of Los Nevados National Natural Park (Colombia) [60]. According to the molecular characterization, this Gram-negative, aerobic, rod-shaped bacterium presented a 99% similarity with the *Serratia proteamaculans* species and, during culture, showed maximum lipolytic activity after 16 h of growth. Optimal growth occurred at 25 °C (growth temperature range between 4 and 30 °C) and at pH 6.0. [60]. In this study, we evaluated the stability features of this new lipase, finding it a very thermophilic lipase. Then, we immobilized the enzyme using both covalent or physical adsorption strategies [61] (Figure 1). In this way, the effect of different immobilization methods on the biocatalytic properties of the lipase USBA-GBX-513 from *Serratia* (ESL) was evaluated. Immobilization methods included: interfacial adsorption on octyl agarose hydrophobic support (OC) [17], covalent binding on octyl glyoxyl Sepharose (OCGLX) after interfacial activation immobilization [44,45,46], direct covalent binding on CNBr-Sepharose [62,63], and anion exchange on Q-Sepharose [61]. The immobilized enzyme derivatives were compared with the commercial lipase B from *Candida antarctica* (CALB), immobilized on octyl Sepharose [18,19,20,21,22,23,24,25,26,27,28,29,30,31,32,33,34,35,36,37,38,39,40,41,42,43,44,45,46,47,48,49,50,51,52,53,54,55,56,57,58,59,60,61,62,63,64]. Very low enzyme loadings of the supports were employed to prevent substrate diffusional limitations [65,66,67,68,69] and to prevent intermolecular enzyme–enzyme interactions that could alter the final performance of the biocatalysts [70,71].

## 2. Results

### 2.1. Immobilization of USBA-GBX-513 Lipase on Different Supports

**Figure 2:**
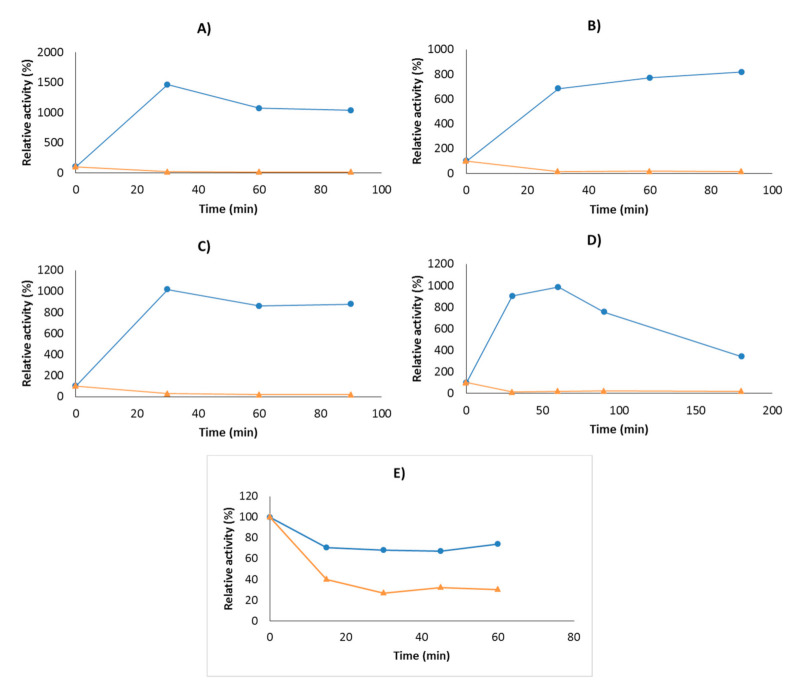
Immobilization courses of USBA-GBX-513 lipase on (**A**) OC-Sepharose; (**B**) OCGLX-Sepharose (**C**) CNBr-Sepharose; (**D**) Q-Sepharose; and (**E**) CALB on OC-Sepharose. The experiments were performed using 1 mg of enzyme/g of support. Other specifications are described in the methods. Orange triangles: supernatant and Blue circles: suspension.

Initially, we studied the immobilization course of the studied lipases on the different supports presented in the introduction. In Figure 2, the immobilization courses of CALB on OC and USBA-GBX-513 lipase on OC-Sepharose, OCGLX-Sepharose, CNBr-Sepharose, and Q-Sepharose supports are depicted. Using USBA-GBX-513 lipase, a hyperactivation effect upon immobilization was observed in all the evaluated supports.

The activity of USBA-GBX-513 lipase immobilized on the OC support was 10.3-fold higher than the activity of the soluble enzyme, which is in good agreement with previous studies using other lipases immobilized on OC-Sepharose supports [44] (Table 1). The covalent immobilization of lipase on OCGLX-Sepharose produced a slight decrease in the enzyme hyperactivation when compared to the just interfacially immobilized lipase (8.2), while the direct covalent enzyme immobilization in CNBr supports increased the enzyme activity by 8.3-fold. Finally, reversible immobilization by ionic interaction of the lipase in Q-Sheparose increased the enzyme activity by 3.4-fold with respect to the activity of the soluble enzyme (Table 1). 

On the other hand, CALB did not exhibit hyperactivation when immobilized on the OC support, as it has been widely reported and consistent with previous studies [44,45,46,64], which is probably due to the fact that this lipase has a small lid that does not completely isolate the active center [72] (Figure 2).

Lipase hyperactivation exhibited after immobilization on hydrophobic supports is caused by lid movements, exposing the active center of the lipase to the medium, which changes the conformational balance between the open and closed form of the lipase [25,26,27,28,29], and fully displaces the equilibrium towards the open active form of the lipase, which is fixed by adsorption on the hydrophobic support through its hydrophobic pocket [17] (Figure 1). This mechanism facilitates the entry of the substrate, resulting in easier enzymatic hydrolysis [16,17,34,73]. In this case, hyperactivation is mediated by the hydrophobic surface, which ensures the lipase immobilization in its open form, avoiding the generation of bimolecular aggregates [22,34,74], and allowing the lipase to exhibit its catalytic activity [16,17,34,44].

With OCGLX, a hetero-functional support, containing glyoxyl and octyl moieties [44] (Figure 1), the lipases are immobilized first via interfacial activation and later via covalent bonds, which can prevent the release of the enzyme under certain reaction conditions [44,45,75]. Covalent immobilization of lipase on CNBr support is performed by a reaction between the most reactive amino group of lipase at pH 7 and the cyanogen bromide group of the support [62,63], maintaining the balance between the open and closed forms of the lipase in a homogeneous medium.

Finally, previous studies have shown that ionic immobilization on Q-Sepharose could retain the hyperactivated enzyme induced by detergents even in the absence of detergents [76]. In this paper, ionic immobilization of the lipase in Q-Sepharose produced lower hyperactivation than the other supports, and detergents have not been used to produce the open form of the lipase. For this reason, we can speculate that improvements in enzyme activity using this support could be related to the breakage of likely enzyme dimers [34] or favorable enzyme distortions caused by the immobilization [16]. 

**Figure 3:**
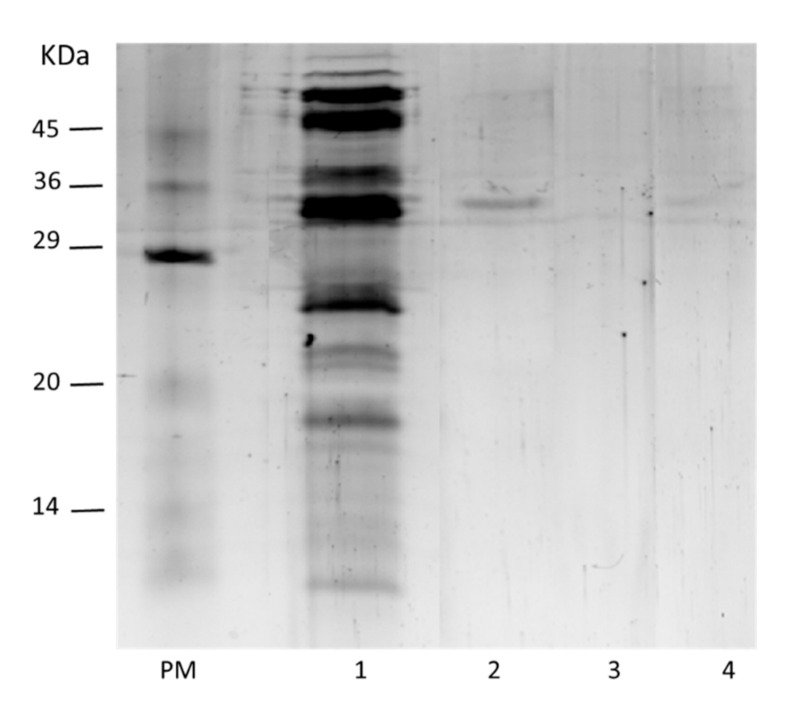
SDS-PAGE analysis of USBA-GBX-513 biocatalyst. PM: Protein Molecular mass marker, Lane 1. Free USBA-GBX-513 extract, Lane 2. OC- USBA-GBX-513, Lane 3. OCGLX- USBA-GBX-513, Lane 4. CNBr-USBA-GBX-513.

Figure 3 shows a gel of the electrophoretic analysis of the lipase initial extract and different immobilized preparations of USBA-GBX-513 lipase from *Serratia* sp. Lipase extract immobilized on OC support evidenced the presence of a single protein band, with a molecular weight of around 34 kDa (lane 2). This indicates that immobilization on this support purifies the lipase in one step, as it has been proved for other lipases [34]. Additionally, it was confirmed that OCGLX-USBA-GBX-513 or CNBr-USBA-GBX-513 irreversibly bound the enzyme to the support, since there was no presence of protein bands (lanes 3 and 4, respectively) in the SDS-PAGE. This covalent immobilization on these supports has already been demonstrated by other authors [44,45,46].

### 2.2. Effect of pH on the Activity of USBA-GBX-513 Lipase Immobilized on Different Supports

**Figure 4:**
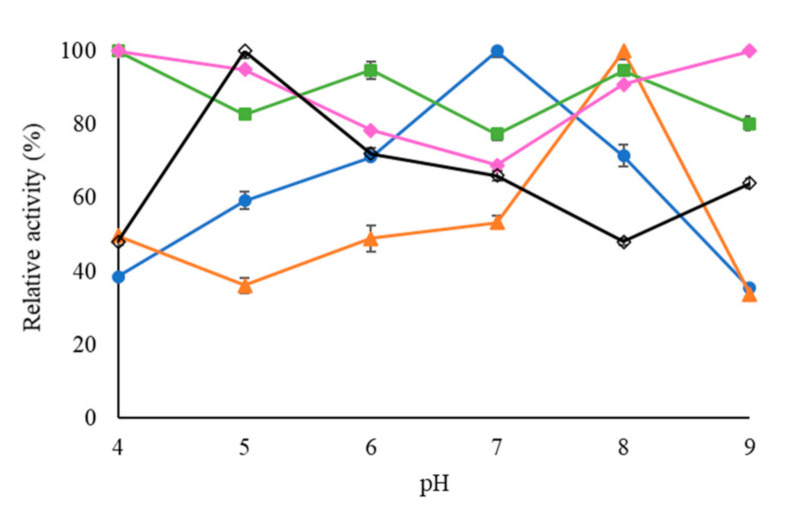
Effect of pH on the activity of different preparations of USBA-GBX-513 lipase on: Empty rhombi: free USBA-GBX-513; blue circles: OC-USBA-GBX-513; orange triangles: OC-GLX-USBA-GBX-513; green squares: Q-Sepharose-USBA-GBX-513; pink rhombi: CNBr- USBA-GBX-513 versus *p*-NPB. Experiments were performed as described in the methods.

Figure 4 shows the activity of the four immobilized derivatives and the soluble form of USBA-GBX-513 lipase at room temperature and a pH range between 4 and 9. This study was performed to check if the enzyme immobilization may alter the response of the enzyme activity to changes in the reaction medium. The free enzyme exhibited the maximum activity in acetate at pH 5, while OC-USBA-GBX-513 showed the highest activity in phosphate at pH 7 and OCGLX-USBA-GBX-513 in carbonate at pH 8. Q-Sepharose-USBA-GBX-513 presented a quite flat profile of activity in all the range of studied conditions. Another important point is that CNBr-USBA-GBX-513 exhibited maximal actives at both of the most extreme studied pH values, 9 and 4, with a minimum at pH 7 (where free OC-USBA-GBX-513 exhibited the maximum activity). It should be considered that the changes in the medium in this study are not only related to the pH value, but also the buffer nature and ionic strength are changed. Nevertheless, the great effect of immobilization on the response of the enzyme to changes in the medium is evident. 

This difference in the effect of pH on the hydrolytic activity of the immobilized derivatives of USBA-GBX-513 lipase fit with previous reports [16] and could be attributed to the involvement of different regions of the lipase surface in the interaction with the support, which generates different enzyme/support interactions, different degrees of rigidity and lipase conformations, etc. However, the differences reported here are higher than those previously reported [16,18,64].

### 2.3. Thermal Stability of USBA-GBX-513 Lipase on Different Supports

**Figure 5:**
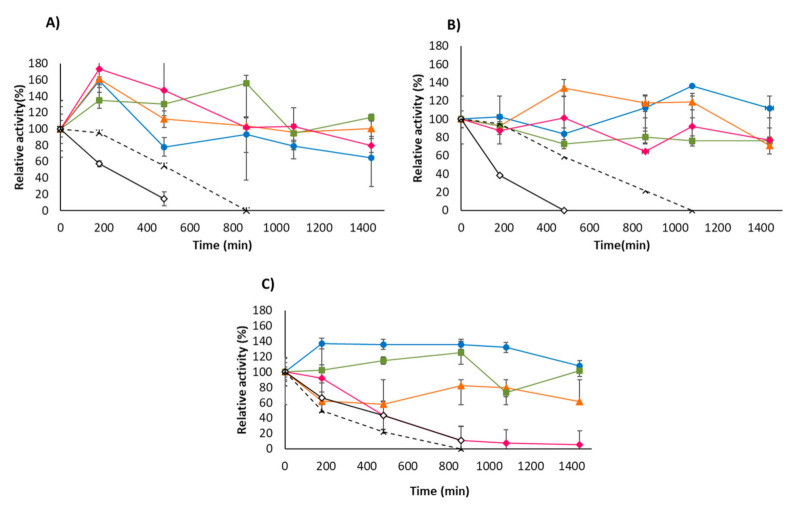
Thermal inactivation at pH (**A**) 5, (**B**) 7, and (**C**) 9 of different USBA-GBX-513 lipase preparations at 90 °C. Empty rhombi: free USBA-GBX-513; dotted line OC-CALB; blue circles: OC-USBA-GBX-513; orange triangles: OCGLX-USBA-GBX-513; green squares: Q-Sepharose-USBA-GBX-513; pink rhombi: CNBr- USBA-GBX-513.

Figure 5 shows that all the immobilized USBA-GBX-513 preparations significantly increased the enzyme stability compared to the free enzyme, an already very stable enzyme. In fact, while the free enzyme is almost fully inactivated during the inactivation time at 90 °C, some of the immobilized USBA-GBX-513 preparations even suffer hyperactivation during this incubation at a high temperature, some just in the initial inactivation times, while other hyperactivations are maintained over time. This increase of the enzyme activity could be related to the thermophilic nature of this enzyme, as many of them suffer a conformational change at high temperatures that makes them more active, presenting an inflexion point in the Arrhenius plot [77,78,79,80]. If the enzyme is stable under the incubation conditions, it can move to a more active conformation at high temperatures, and the measure of the activity may detect these more active lipase forms, as the reversion of these changes should not be instantaneous at 25 °C (we found a similar inactivation course when incubating the biocatalysts at 25 °C for 5 min before adding the substrate).

With this hyperactivation, it is not simple to state which is the most stable biocatalyst. At pH 5, it seems that OC-USBA-GBX-513 is slightly less stable than the other biocatalysts, while it is the most stable at pH 7 and 9. The literature shows that lipases immobilized via interfacial activation on hydrophobic supports become highly stabilized, because lipases in the stabilized open form are more stable than the lipase in the conformational equilibrium [81,82,83]. In fact, lipases immobilized using this strategy tend to be even more stable than the lipase immobilized via multipoint covalent attachment [84,85,86,87].

The promotion of covalent bonds on this derivative, using OCGLX-USBA-GBX-513 [44], presented a negative effect on enzyme stability. Although, in general, the covalent bonds obtained using this heterofunctional support increase enzyme stability, in some instances, it has been proved to be slightly negative for enzyme stability, even though this will prevent enzyme release [44]. This was explained by the distortions and tensions caused in the enzyme structure by the promotion of the covalent bonds.

CNBr-USBA-GBX-513 presented a stability similar to that of the free enzyme at pH 9, but it was significantly more stable at pH 5 and 7 than the free enzyme. It should be expected that the limited formation of enzyme-support covalent bonds could not significantly increase the enzyme rigidity, therefore the stabilization at pH 5 and 7 could be related to the suppression of some intermolecular process (e.g., aggregation) after enzyme immobilization in porous supports [5].

Finally, the enzyme immobilized via anionic exchange showed an intermedium stability under all the studied pH values. The immobilization via ion exchange may produce almost any result in terms of enzyme stability, as the support generates an enzyme nanoenviroment that may be positive or negative for the enzyme stability depending on the circumstances [40].

The fact that the most stable biocatalyst depends on the inactivation conditions agrees with previous reports stating that the enzyme inactivation pathway may be different depending on the inactivation conditions [87].

OC-CALB, used as a very stable biocatalyst of one of the most used enzymes [14,15,18,19,64,88,89], was the least stable biocatalyst, even with a lower stability than free USBA-GBX-513. This exemplifies the great advantage of the use of thermophilic enzymes [53,54,55,56,57,58,59].

### 2.4. Stability of the Immobilized USBA-GBX-513 and CALB in the Presence of Organic Solvents

**Figure 6:**
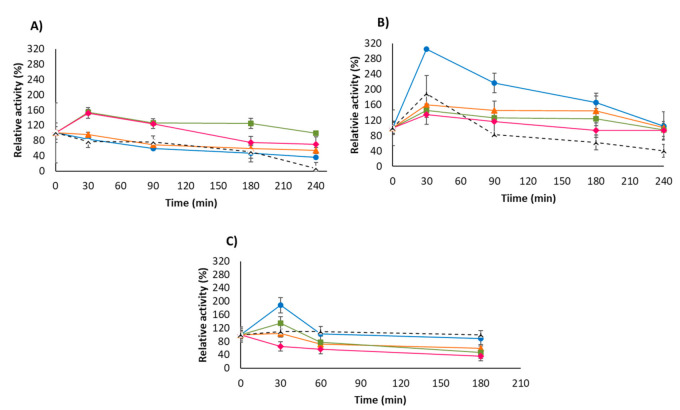
Stability in organic cosolvents of different lipase preparations: (**A**) ACN, (**B**) THF, and (**C**) 1,4-dioxane. Experiments were performed at 50% (*v*/*v*) in 50 mM Tris-HCl pH 7 and 40 °C. Dotted line OC-CALB; blue circles: OC-USBA-GBX-513; orange triangles: OC-GLX-USBA-GBX-513; green squares: Q-Sepharose- USBA-GBX-513; pink rhombi: CNBr- USBA-GBX-513.

The stability in organic solvents of USBA-GBX-513 derivatives was investigated in the presence of 50% (*v*/*v*) 1,4-dioxane, acetonitrile (ACN), or tetrahydrofuran (THF) at 40 °C (Figure 6).

The enzyme activity of some preparations showed some initial increases, and in general, very high activity levels were maintained for all preparations during the inactivation times. This increase in enzyme activity should be explained by some positive conformational changes induced by these incubation conditions. 

The highest hyperactivation was found using THF and OC-USBA-GBX-513. This preparation was also the one that suffers the highest hyperactivation in 1,4-dioxane. After the initial hyperactivation, this preparation showed a slow decrease in its activity. However, using ACN, this biocatalyst showed the lowest residual activity. OCGLX- USBA-GBX-513 was the least stable biocatalyst in THF and 1,2 dioxane, and similar to the reversibly immobilized OC-USBA-GBX-513 in ACN, suggesting that enzyme release from the OC support was not a significant cause of the enzyme inactivation.

The other two immobilized preparations were the most stable in ACN, while they were the least stable ones in the other two solvents. That is, depending on the solvent, the most and the least stable enzyme biocatalysts changed, again suggesting different responses of the immobilized enzymes to the medium depending on the immobilization protocol.

OC-CALB stability in organic media was not so different to the stability of the USBA-GBX-513 biocatalyst; in fact, in 1-4 dioxane, it was even the most stable one, although in the other two solvents, it was the least stable. In any case, the differences were not as significant as in the thermal inactivation studies (Figure 5). This may be a possibility since although the enzyme USBA-GBX-513 has evolved to be more thermostable, this may not be necessarily translated to a higher stability under other inactivation conditions. Even if some papers show some correlation between the thermophilicity of the enzyme producer and stability in organic solvents [21,59,90], this may not be a universal rule.

## 3. Materials and Methods

### 3.1. Materials

Non-commercial extract of extremophilic lipase USBA-GBX-513 and commercial lipase B from *Candida antarctica*, CALB from Novozymes (Madrid, Spain) were used. Octyl Sepharose (OC) 4BCL, Q-Sepharose 4B fast flow, cyanogen bromide-activated-Sepharose 4B (CNBr-Sepharose), *p*-nitrophenyl butyrate (*p*-NPB), ammonium acetate, ammonium phosphate, sodium acetate, sodium bicarbonate, Tris-HCl, sodium borohydride, ethanolamine, sodium periodate, potassium iodide, tetrahydrofuran, 1,4-dioxane, and acetonitrile were purchased from Sigma Chemical Co. (St. Louis, MO, USA). All reagents and solvents were of analytical grade. Octyl-glyoxyl (OCGLX) support was prepared from OC as previously described [44]. 

### 3.2. Strains and Growth Conditions for Enzyme Production

*Serratia* sp. USBA-GBX-513 was isolated from paramo soils in Los Nevados National Natural Park (Colombia) deposited in the USBA collection of microorganisms with the code CMPUJ U513. Lipase was produced by submerged fermentation. Bacterial inoculums were produced in LB medium. Microbial cultures were started by inoculating *Serratia* sp. USBA-GBX-513, obtaining an OD600 around 0.150 using LB medium and 0.1% (*v*/*v*) olive oil for induction of lipase production and incubated at 30 °C and 180 rpm. Subsequently, after 72 h of fermentation, microbial cells were recovered by centrifugation (8000 rpm for 10 min); and the pellet was recovered, washed three times by successive centrifugations at 6000 rpm for 30 min at 4 °C, using 0.85% (*w*/*v*) NaCl to re-suspend the pellets; and finally frozen at −20 °C for a period of 48 h. After this time, the pellet was re-suspended in 50 mM sodium phosphate at pH 7.5, and submitted to ultrasounds to force the cells lysis using the following parameters: amplitude: 40%, total time: 10 min, pulses on: 20 s and pulses off: 30 s. Then, the suspension was centrifuged at 8000 rpm for 10 min and the supernatant was collected and used without further purification.

### 3.3. Standard Measure of Enzyme Activity

The enzyme activity of the soluble and immobilized enzyme was determined by the hydrolysis of *p*-NPB. The concentration of released *p-*nitrophenol was quantified spectrophotometrically at 348 nm. An initial 50 mM *p*-NPB solution was prepared in acetonitrile. Activity measurements were performed in 25 mM sodium phosphate solution at pH 7.0 and 25 °C, containing 1 mM *p*-NPB (50 μL of *p*-NPB solution) (ε = 5150 mol/cm under these conditions) [12,51,54], using a volume of 2.5 mL of buffer. To initiate the reaction, 25–100 μL of suspended lipase or solution was added. An international unit of activity (U) was defined as the amount of enzyme that hydrolyses 1 μmol of *p*-NPB per minute under the conditions described above. Protein concentration was measured by the Bradford method using bovine serum albumin (BSA) as standard protein [91].

### 3.4. Immobilization of Enzymes on Octyl (OC) and Octyl-Glyoxyl (OCGLX) Supports

Immobilization was performed using 1 mg of protein per g of wet support. Enzymes were diluted in the corresponding volume of 5 mM sodium phosphate at pH 7 and 25 °C under stirring at 210 rpm, and then, the indicated support was added to this enzymatic solution (1 g of support per 10 mL of enzyme solution). Enzyme activities in both supernatant and suspension solutions were followed using the *p*-NPB assay (see above). After enzyme immobilization, the different immobilized enzyme biocatalysts were recovered by filtration and washed several times with distilled water. To prepare OCGLX-lipase biocatalyst, the immobilized enzyme (obtained as the OC preparation) was re-suspended in 50 mM NaHCO_3_/NaOH at pH 10.05 for 16 h at 25 °C, to favor the covalent attachment of the enzyme onto the support [44,45]. Finally, solid sodium borohydride (NaBH₄) was added to reach a concentration of 1 mg/mL, and the suspension was submitted to stirring at 150 rpm for 30 min in an ice bath to reduce Schiff base linkages between the immobilized enzyme and the support. Finally, the reduced derivatives were filtered, washed with abundant distilled water, and stored at 4 °C [44,45,46].

### 3.5. Immobilization of Lipase onto CNBr-Sepharose Support

Cyanogen bromide-activated-Sepharose 4B (CNBr-Sepharose) was hydrated in 1 mM HCl (pH 2.5) under stirring at 40 rpm for 15 min (1 g of support per 10 mL of HCl solution). Then, the immobilization was performed using 1 mg of protein per g of wet support. The enzymes were diluted in the corresponding volume of 25 mM sodium phosphate at pH 7 and 25 °C under stirring at 210 rpm for 90 min. Periodically, samples of the supernatant and suspension were withdrawn, and the enzyme activity was measured as described above. After immobilization, immobilized biocatalysts were washed with distilled water and incubated in a 1 M solution of ethanolamine, at pH 8 and 4 °C for 20 h, in order to block remaining reactive groups in the support. Finally, these immobilized preparations were washed with abundant distilled water and stored at 4 °C [17,70]. 

### 3.6. Immobilization of Lipase on Q-Sepharose Support

Immobilization of lipases on the anion exchanger support was performed using 1 mg of protein per gram of Q-Sepharose dissolved in 5 mM sodium phosphate at pH 7 (1 g of support per 10 mL of enzyme solution). The immobilization suspension was stirred at 25 °C and 210 rpm for 3 h. Periodically, samples of the supernatants and suspensions were withdrawn, and the enzyme activity was measured as described above. After that, the supported lipase was recovered by filtration and washed several times with distilled water [92,93].

### 3.7. SDS-PAGE Analysis

SDS-polyacrylamide gel electrophoresis was performed according to Laemmli [94], using a Miniprotean tetra-cell (Biorad), 12%(*w*/*v*) running gel in a separation, and a concentration zone of 4% (*w*/*v*) polyacrylamide. Ten milligrams of the immobilized enzyme samples were re-suspended in 60 µL of rupture buffer (2 % (*v*/*v*) SDS and 10% (*v*/*v*) mercaptoethanol), boiled for 5 min, and a 25-µL aliquot of the supernatant was used in the experiments. This treatment ensures the release of the enzyme molecules from the support [44]. Gels were stained with Coomassie brilliant blue. 

### 3.8. Effect of pH on Enzyme Activity

Enzyme activity was measured using *p*-NPB at pH 4–9, as described previously, but using 25 mM of the different buffers (sodium acetate at pH 4–6, Tris-HCl at pH 7, and sodium carbonate at pHs 8 and 9). The solution of *p*-NPB was added 10 min after adding the enzyme to the respective buffer to ensure the pH equilibration in the core of the biocatalyst particle [16].

### 3.9. Thermal Inactivation 

Thermal stability of the different enzyme preparations was determined by re-suspending 40 mg of the immobilized enzyme in 2 mL of 50 mM sodium acetate at pH 5, Tris-HCl at pH 7, or sodium bicarbonate at pH 9 at 90 °C. Periodically, samples were withdrawn, and the activity was measured using *p*-NPB as described above.

### 3.10. Solvent Inactivation 

Different enzyme preparations were incubated in mixtures of 50% (*v*/*v*) of acetonitrile (ACN), 1,4-Dioxane or tetrahydrofuran (THF)/50% 50 mM Tris-HCl, at pH 7 and 40 °C. Periodically, samples were withdrawn, and the activity was measured using *p*-NPB as described above. Free enzyme was not included due to the possibility of enzyme aggregation, which produces confusing results [5,16].

## 4. Conclusions

The USBA-GBX-513 enzyme was produced by a microorganism isolated from a psychrophilic environment. This enzyme presented the ability to withstand extreme thermal conditions (90 °C) according to the data reported in this study. It was found that the enzyme immobilization on different types of supports and using different immobilization mechanisms (limited-point covalent union, ionic exchange, interfacial activation, and this last one combined with limited covalent attachment) caused hyperactivation and stabilized USBA-GBX-513.

Although all immobilized preparations were more stable than the free enzyme, interestingly, the most stable biocatalyst depends on the inactivation conditions. Q-sepharose-USBA-GBX-513 was the most stable derivative at pH 5, while OC-USBA-GBX-513 was the most stable biocatalyst at pH 7 and 9. This change of the most stable biocatalysts with the inactivation conditions is evidence of the interest of generating a battery of biocatalysts of this enzyme to find its optimal biocatalyst. In thermal inactivation, all USBA-GBX-513 clearly enhanced the stability of OC-CALB.

In the presence of organic solvents, OC-USBA-GBX-513 presented the highest retention of activity in the presence of solvents, such as THF and 1,4-dioxane, but was the least stable in the presence of ACN. In organic solvents, the differences with OC-CALB were not so evident. Again, the immobilization protocol deeply affected the response of the enzyme to changes in the medium.

In conclusion, immobilization on different supports of this lipase from a psychrophilic microorganism allowed a remarkable improvement of the enzyme properties, such as the activity and thermal and solvent stabilities under different pH values, which enlarges their applications under different environmental reaction conditions. The presented studies were performed using a model synthetic substrate without real applicability. The actual applicability of the biocatalysts prepared in this paper should be checked with the specific substrates involved in the different future processes where this interesting enzyme may be utilized, and under the specific reaction conditions. This will be matter of further investigation in our research group.

## Figures and Tables

**Table 1:** Hyperactivation values during immobilization of USBA-GBX-513 lipase on different activated supports.

Support	Hyperactivation Factor (Fold)
OC	10.3
OCGLX	8.2
CNBr-Sepharose	8.8
Q-sepharose	3.4

## Data Availability

All data are presented in the paper.

## Research and Genetic Resources Access Permits

This research was funded by the contract MAVDT No. 15, 2008 for genetic resources access (GRA) and the research authorization No. DTNO-N-20/2007.

## Sample Availability

Samples of the enzyme and immobilized enzymes are available from the authors.
